# Supplementary material for: Nutrient and arsenic biogeochemistry of Sargassum in the western Atlantic
Source: Nat Commun. 2023 Oct 5;14:6205. doi: 10.1038/s41467-023-41904-4 (PMC10556059; doi:10.1038/s41467-023-41904-4)
Supplement: Supplementary file 1 — Supplementary information [file 41467_2023_41904_MOESM1_ESM.pdf]

Nutrient and arsenic biogeochemistry of *Sargassum* in the western Atlantic

Dennis Joseph McGillicuddy, Jr.[1]
Peter Lynn Morton[2]
Rachel Aileen Brewton[3]
Chuanmin Hu[4]
Thomas Bryce Kelly[5]
Andrew Robert Solow[1]
Brian Edward Lapointe[3]

Supplementary Information

September 18, 2023

[1]Woods Hole Oceanographic Institution, Woods Hole, MA, USA.
*Corresponding Author. dmcgillicuddy@whoi.edu
[2]Department of Oceanography, Texas A&M University, College Station, TX, USA.
[3]Harbor Branch Oceanographic Institute, Florida Atlantic University, Fort Pierce, FL, USA.
[4]College of Marine Science, University of South Florida, St. Petersburg, FL, USA.
[5]College of Fisheries and Ocean Science, University of Alaska Fairbanks, Fairbanks, AK, USA.

Supplementary Table 1. Subregional comparisons of elemental composition (% dry weight) and ratios, as well as stable isotope values (‰) for *S. fluitans* and *S. natans*. Mean and 95% confidence interval are indicated in each case. See Fig. 1 for geographic boundaries of the subregions.

	Northern Sargasso Sea		Sargasso Sea		Caribbean		Western Tropical Atlantic	
	S. fluitans	*S. natans*	*S. fluitans*	*S. natans*	*S. fluitans*	*S. natans*	*S. fluitans*	*S. natans*
N	6	12	20	43	30	29	30	30
%C	25.2 ± 1.5	27.5 ± 1.7	29.5 ± 0.9	30.0 ± 1.0	26.3 ± 0.6	25.6 ± 0.6	27.5 ± 0.5	27.2 ± 0.8
%N	2.0 ± 0.5	2.0 ± 0.3	0.8 ± 0.1	0.8 ± 0.1	1.3 ± 0.1	1.4 ± 0.1	1.1 ± 0.1	1.3 ± 0.1
%P	0.21 ± 0.04	0.19 ± 0.03	0.08 ± 0.01	0.07±0.01	0.12 ± 0.01	0.15 ± 0.02	0.14 ± 0.02	0.15 ± 0.02
C:N	15.6 ± 4.3	16.9 ± 3.2	45.1 ± 6.7	52.3 ± 6.4	25.1 ± 1.8	22.0 ± 1.7	30.4 ± 3.3	26.2 ± 2.0
C:P	320 ± 63	419 ± 100	984 ± 142	1363 ± 179	619 ± 74	469 ± 49	570 ± 71	506 ± 67
N:P	21.0 ± 3.4	24.7 ± 2.7	23.9 ± 4.8	26.9 ± 2.9	24.4 ± 1.7	21.6 ± 2.0	18.9 ± 1.6	19.2 ± 1.6
$\delta^{15}N$	1.93 ± 0.58	1.82 ± 0.18	-0.95 ± 0.36	-1.40 ± 0.34	-0.71 ± 0.41	-0.71 ± 0.37	-0.77 ± 0.46	-1.22 ± 0.40
%As	0.010 ± 0.005	0.012 ± 0.003	0.015 ± 0.002	0.016 ± 0.002	0.009 ± 0.001	0.008 ± 0.0004	0.010 ± 0.001	0.010 ± 0.001

Supplementary Table 2. Statistical significance (p-values of two-sided t-tests) for subregional comparisons of mean elemental composition, stoichiometry, and $\delta^{15}N$ stable isotope values for the combined species *S. fluitans* and *S. natans*. Cells with p-values in excess of 0.05 are highlighted. See Fig. 1 for geographic boundaries of sample groupings in the Northern Sargasso Sea (NSS), Sargasso Sea (SS), Caribbean (CAR), and Western Tropical Atlantic (WTA).

**%C**	SS	CAR	WTA	**%N**	SS	CAR	WTA	**%P**	SS	CAR	WTA
NSS	1.03e-04	0.21	0.36		2.40e-10	2.94e-06	1.79e-07		2.53e-09	7.82e-05	7.19e-04
SS		6.94e-15	1.08e-07			3.88e-25	4.20e-16			1.56e-16	2.91e-18
CAR			1.33e-05				9.80e-04				0.17
**C:N**				**C:P**				**N:P**			
NSS	1.10e-20	4.42e-06	2.31e-10		3.87e-18	3.55e-04	6.10e-04		0.11	0.74	6.72e-04
SS		2.65e-16	1.29e-12			7.24e-15	4.68e-15			0.04	1.46e-06
CAR			1.03e-04				0.83				1.24e-05
**%As**				**As:C**				**As:N**			
NSS	2.73e-03	5.38e-03	0.09		0.02	4.50e-03	0.04		1.12e-13	0.68	0.01
SS		1.27e-16	9.99e-13			4.01e-16	3.36e-12			6.11e-14	4.70e-12
CAR			9.62e-06				3.67e-03				4.03e-06
**As:P**				**$\delta^{15}N$**							
NSS	6.58e-14	0.74	0.48		2.74e-31	1.91e-25	1.59e-26				
SS		2.28e-14	4.74e-14			3.88e-03	0.19				
CAR			0.49				0.16				

Supplementary Table 3. Summary of least squares fits $y = ax + b$ for arsenic content as a function of phosphorus, nitrogen, and carbon content (% dry weight). Asterisk indicates p-values so low that they could not be distinguished from zero.

y	x	r^2	Intercept (b)	p	Slope (a)	p
As	P	0.23	1.60e-02	0.00e+00*	-3.79e-02	1.16e-12
As	N	0.27	1.75e-02	0.00e+00*	-5.25e-03	3.66e-15
As	C	0.50	-1.98e-02	3.33e-16	1.13e-03	0.00e+00*

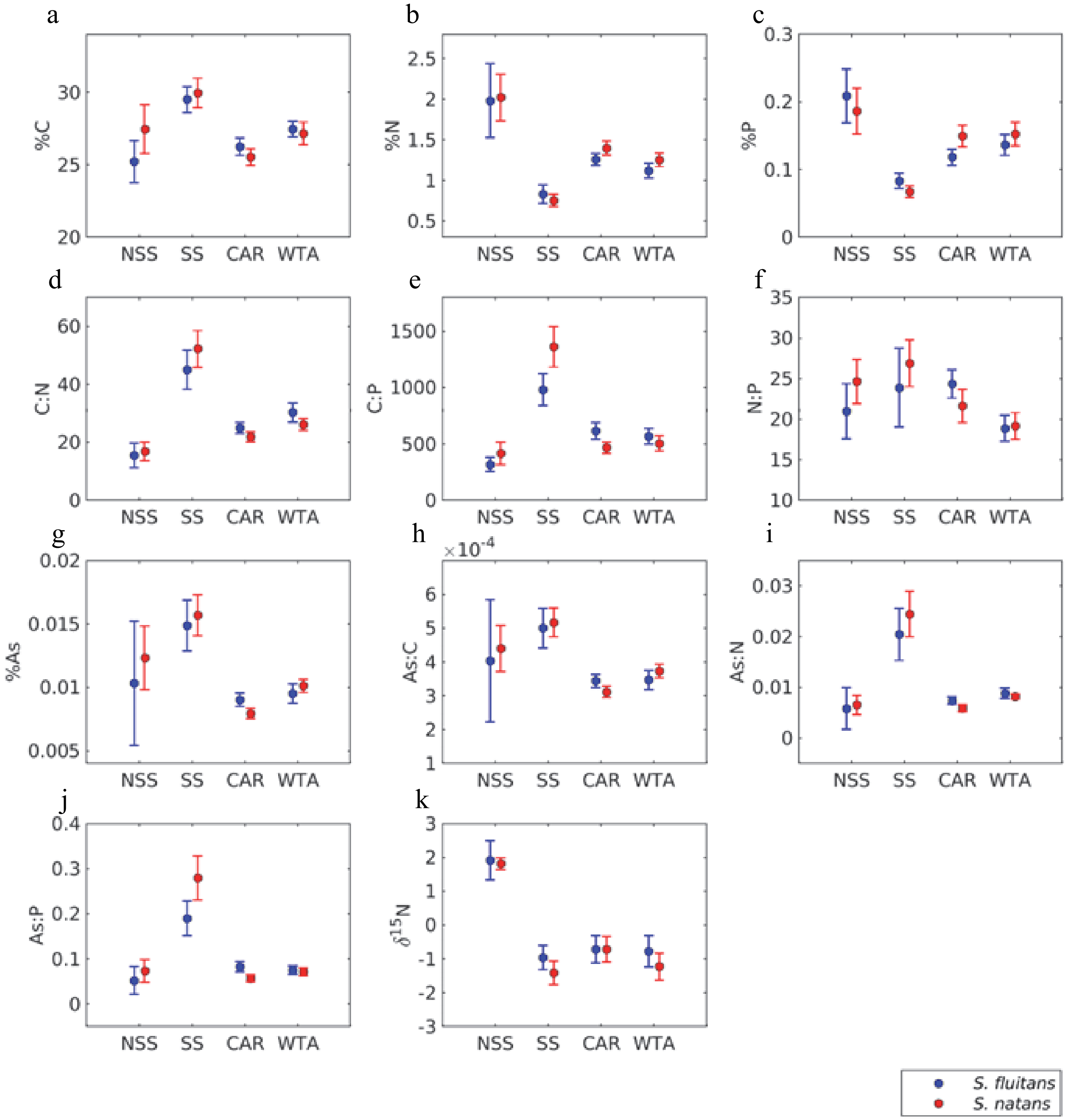

Supplementary Fig. 1. **Subregional comparisons (by species) of elemental content, ratios, and stable isotope values for the Northern Sargasso Sea (NSS), Sargasso Sea (SS), Caribbean (CAR), and Western Tropical Atlantic (WTA).** **a** Carbon content. **b** Nitrogen content. **c** Phosphorus content. **d** Carbon to nitrogen. **e** Carbon to phosphorus. **f** Nitrogen to phosphorus. **g** Arsenic content. **h** Arsenic to carbon. **i** Arsenic to nitrogen. **j** Arsenic to phosphorus. **k** $\delta^{15}N$. Error bars are 95% confidence intervals.

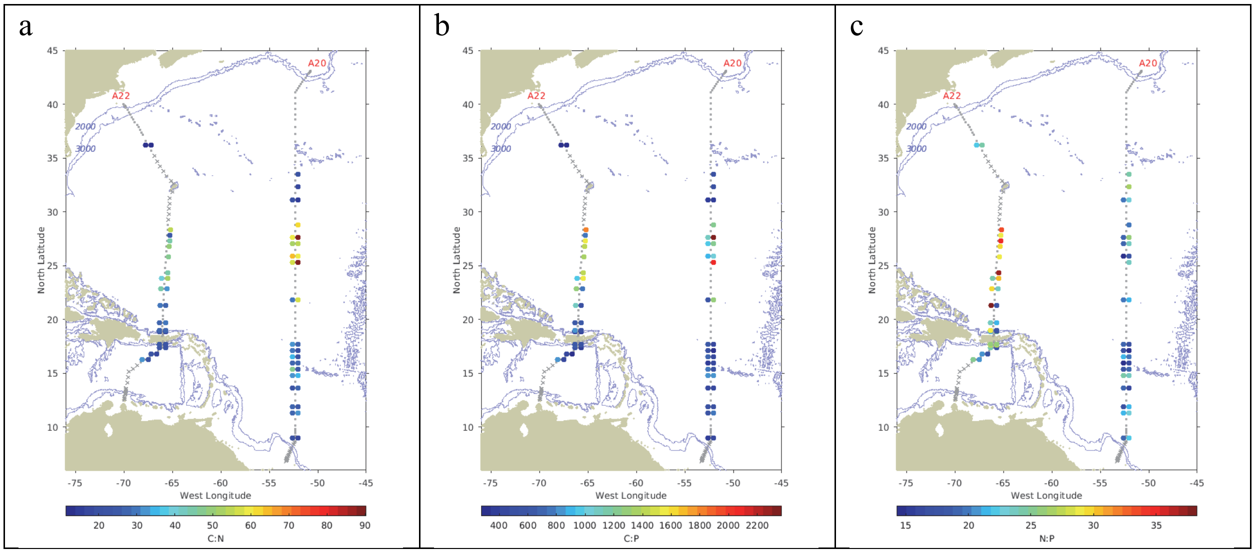

Supplementary Fig. 2. ***Sargassum* elemental ratios. a** Carbon to nitrogen. **b** Carbon to phosphorus. **c** Nitrogen to phosphorus. *S. fluitans III* is shown to the left of transects A20 and A22, and *S. natans I* to the right. Stations where no *Sargassum* was found are shown as circles, and stations where *Sargassum* sampling was not possible are shown as Xs. Blue lines are the 2000 m and 3000 m isobaths.

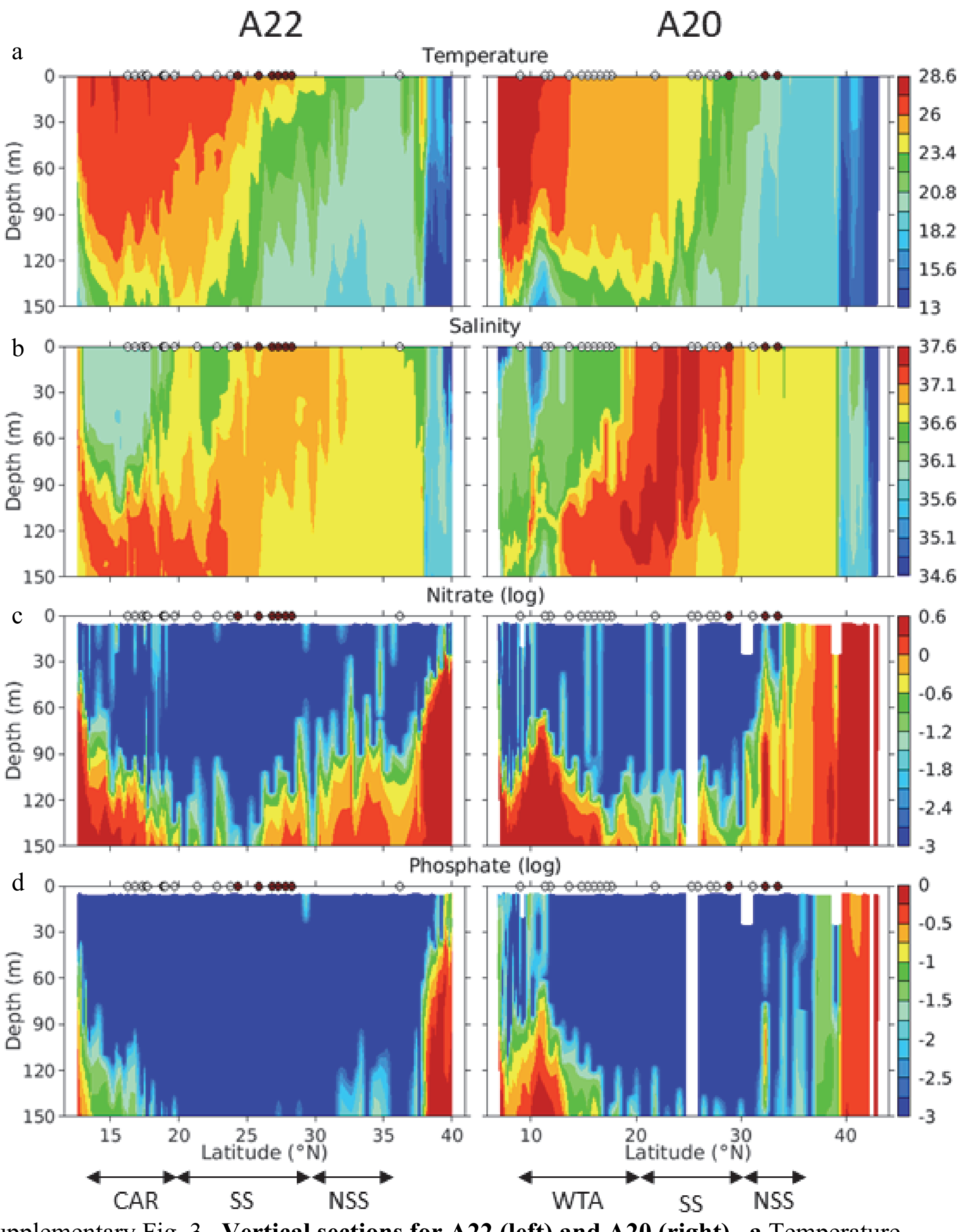

Supplementary Fig. 3. **Vertical sections for A22 (left) and A20 (right).** **a** Temperature. **b** Salinity. **c** Nitrate. **d** Phosphate. Nitrate and phosphate concentrations (µmol kg^{-1}) have been log transformed in order to better resolve gradients at low concentrations. Circles along the top of each plot indicate stations where *Sargassum* was collected: *S. fluitans* and *S. natans* (white) and *S. natans* only (brown). Northern Sargasso Sea (NSS), Sargasso Sea (SS), Caribbean (CAR), and Western Tropical Atlantic (WTA) regions are indicated.

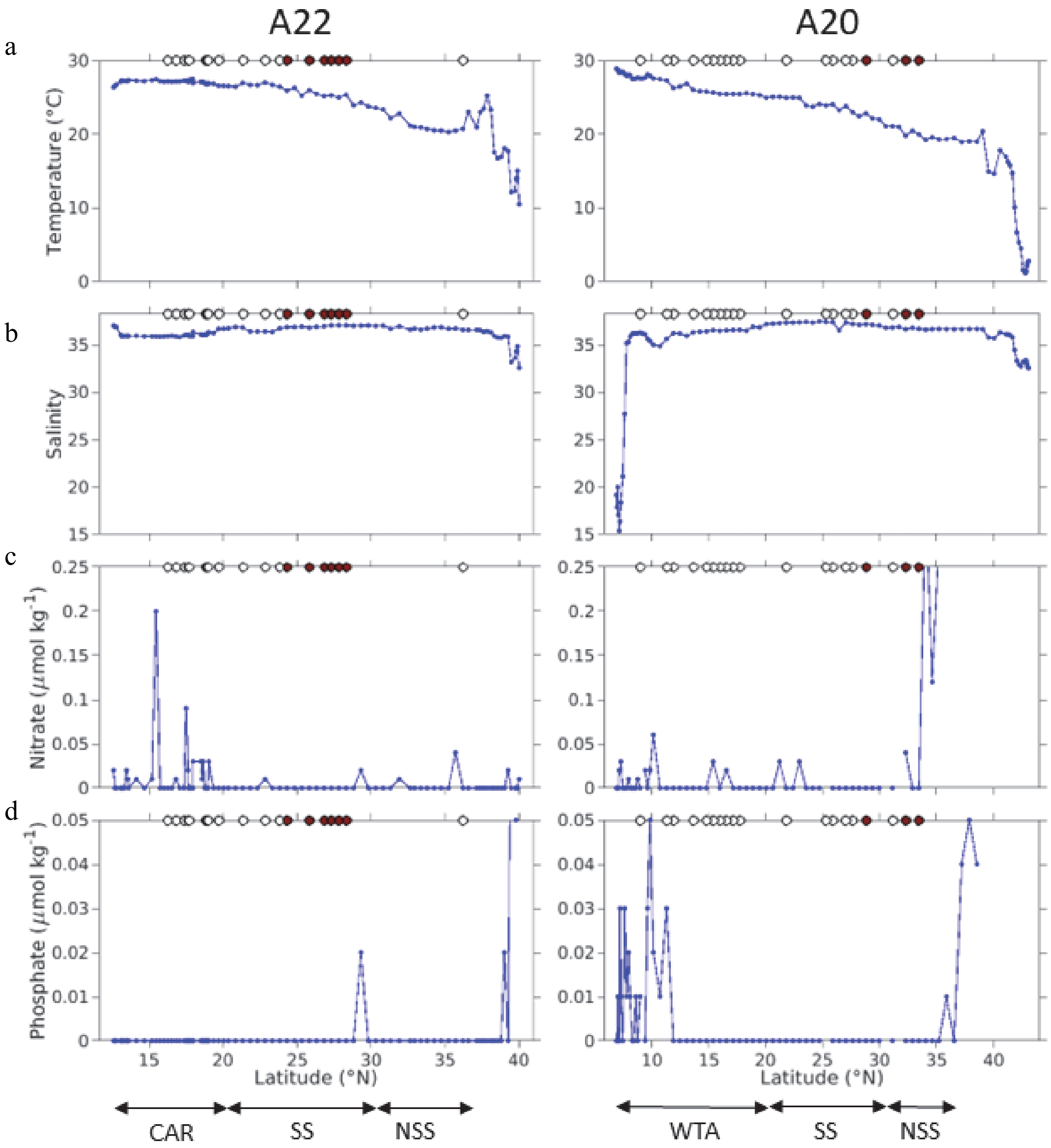

Supplementary Fig. 4. **Surface properties for A22 (left) and A20 (right). a** Temperature. **b** Salinity. **c** Nitrate. **d** Phosphate. Circles along the top of each plot indicate stations where *Sargassum* was collected: *S. fluitans* and *S. natans* (white) and *S. natans* only (brown). Northern Sargasso Sea (NSS), Sargasso Sea (SS), Caribbean (CAR), and Western Tropical Atlantic (WTA) regions are indicated.

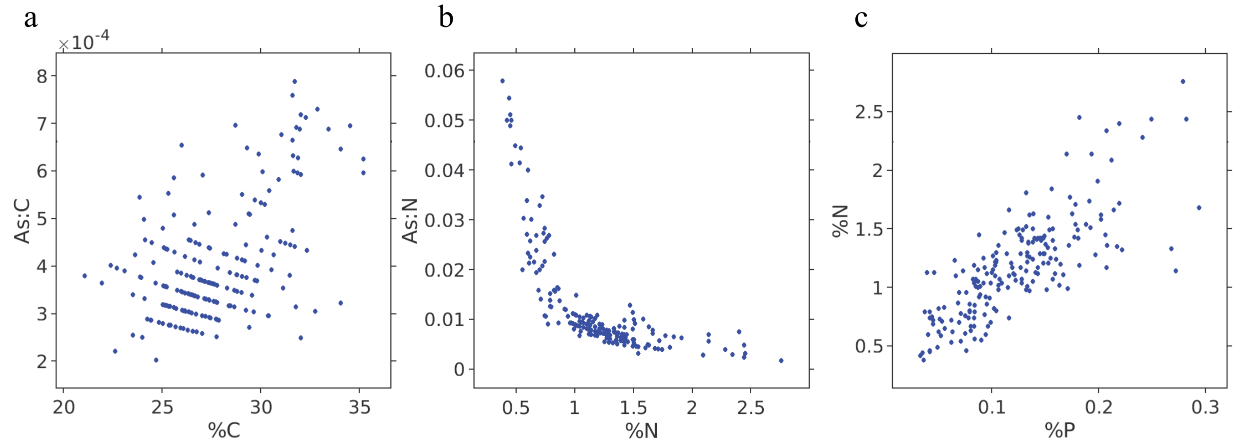

Supplementary Fig. 5. **Elemental ratios in *Sargassum* tissue.** **a** Arsenic to carbon ratio as a function of carbon content. **b** Arsenic to nitrogen ratio as a function of nitrogen content. **c** Nitrogen content as a function of phosphorus content.

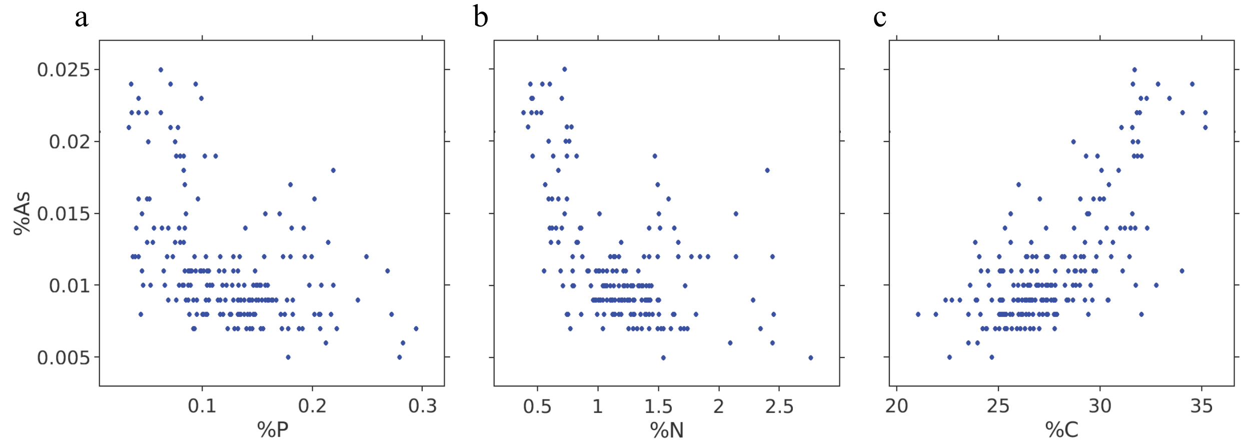

Supplementary Fig. 6. **Arsenic in *Sargassum* tissue.** **a** Arsenic content as a function of phosphorus content. **b** Arsenic content as a function of nitrogen content. **c** Arsenic content as a function of carbon content. Results of least squares fits are presented in Supplementary Table 3.

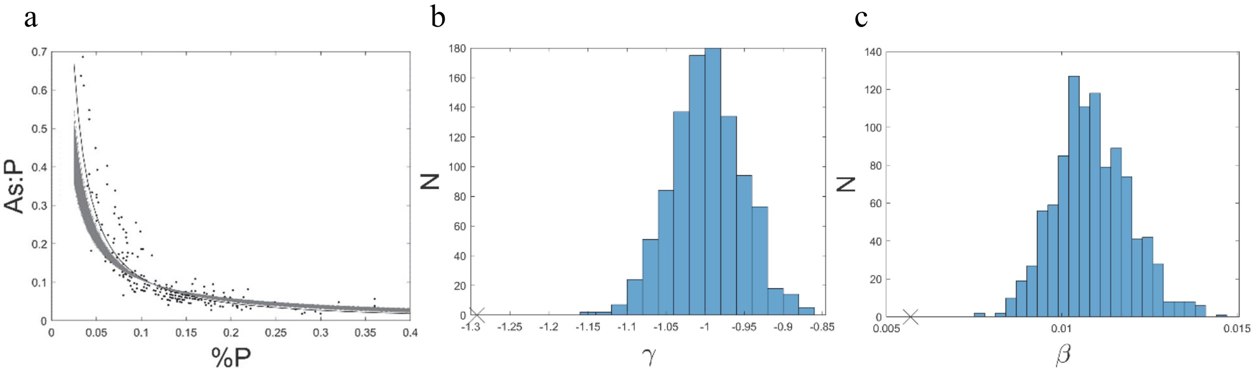

Supplementary Fig. 7. **Evaluation of the model for the arsenic to phosphorus ratio in *Sargassum* tissue. a** Fits of the arsenic to phosphorus ratio in *Sargassum* ratio as a function of phosphorus content. Solid line is the fit to the data from Fig. 4 (black dots), and the gray lines are 1000 trials using the permuted phosphorus data as described in the Supplemental Note. **b,c** The distribution of parameters γ and β for the permuted data (blue bars) and the actual data (X on the x-axes).

Supplementary Note

The general model relating observations i of arsenic and phosphorus content we wish to test is

$$\frac{As_i}{P_i} = \beta P_i^{\gamma} \tag{1}$$

Where $\gamma = -1$ would constitute the $\frac{1}{P}$ dependence predicted by the theory. This model can be recast as

$$log(As_i) = c + (\gamma + 1)log(P_i) \tag{2}$$

where $c = log(\beta)$. The parameters c and γ were estimated by least squares, and it turns out that the dependence of As:P on P is supra-hyperbolic: $\gamma = -1.29 \pm 0.04$. We tested the statistical robustness of this finding by permuting the independent variable P_i in Equation (2) 1000 times, and the fit to the actual data falls outside that envelope (Supplementary Fig. 7). Thus, we conclude our result is robust with p<0.001.

We note that recent observations suggest variation of As content among genotypes, with *S. natans VIII* having higher As content than *S. natans I* and *S. fluitans III*[1,2]. Because our methods did not distinguish *S. natans VIII*, it would have been grouped together with *S. fluitans III* due to their morphological similarity. Our data do not indicate large variations in As content among the species we resolved (Supplementary Fig. 1, Supplementary Table 1), so this analysis was based on the As content of the combined species.

Supplementary References

1 Cipolloni, O.-A. *et al.* Metals and metalloids concentrations in three genotypes of pelagic *Sargassum* from the Atlantic Ocean Basin-scale. *Marine Pollution Bulletin* **178**, 113564 (2022). https://doi.org:https://doi.org/10.1016/j.marpolbul.2022.113564

2 Gobert, T. *et al.* Trace metal content from holopelagic *Sargassum* spp. sampled in the tropical North Atlantic Ocean: Emphasis on spatial variation of arsenic and phosphorus. *Chemosphere* **308**, 136186 (2022). https://doi.org:https://doi.org/10.1016/j.chemosphere.2022.136186
